# Evaluation of Hydroxychloroquine Blood Concentrations and Effects in Childhood-Onset Systemic Lupus Erythematosus

**DOI:** 10.3390/ph14030273

**Published:** 2021-03-17

**Authors:** Noël Zahr, Saik Urien, Christian Funck-Brentano, Hélène Vantomme, Nicolas Garcelon, Isabelle Melki, Margaux Boistault, Olivia Boyer, Brigitte Bader-Meunier

**Affiliations:** 1 Clinical Investigation Center, Department of Pharmacology, INSERM, CIC-1901, UMR ICAN 1166, Pitié-Salpêtrière Hospital, Sorbonne Université, AP-HP, F-75013 Paris, France; christian.funck-brentano@aphp.fr (C.F.-B.); helene.vantomme@aphp.fr (H.V.); 2 Department of Pediatric and Perinatal Pharmacology, Necker Hospital, Université de Paris, AP-HP, F-75015 Paris, France; saik.urien@aphp.fr; 3 Data Science Platform, INSERM UMR 1163, Imagine Institute, Université de Paris, AP-HP, F-75015 Paris, France; nicolas.garcelon@institutimagine.org; 4 Infectious Disease and Internal Medicine Reference Center for Rheumatic, AutoImmune and Systemic Diseases in Children (RAISE), Department of General Pediatrics, Robert Debré Hospital, Nord-Université de Paris, AP-HP, F-75019 Paris, France; isabelle.melki@aphp.fr; 5 Reference Center MARHEA, Department of Pediatric Nephrology, Imagine Institute, INSERM U1163, Necker Hospital, Université de Paris, AP-HP, F-75015 Paris, France; Margaux.boistault@aphp.fr (M.B.); olivia.boyer@aphp.fr (O.B.); 6 Department of Pediatric Immunology, Hematology and Rheumatology, INSERM U1163, Imagine Institute, Necker Hospital, Université de Paris, AP-HP, F-75015 Paris, France; brigitte.bader-meunier@aphp.fr

**Keywords:** hydroxychloroquine, systemic lupus erythematosus, pharmacokinetics, pharmacodynamics

## Abstract

Background: Hydroxychloroquine (HCQ) is an antimalarial agent given to patients with systemic lupus erythematosus (SLE) as first-line therapy. It alleviates childhood-onset systemic lupus erythematosus cSLE skin and musculoskeletal disease, decreasing disease activity and flares. HCQ concentration–effect relationships in children remains unknown. This study aimed to investigate the pharmacokinetics of HCQ and possible concentration–effect relationships. Methods: HCQ blood concentrations and effects were obtained during clinical follow-up on different occasions. cSLE flares were defined using the SLE Disease Activity Index (SLEDAI); flare was denoted by a SLEDAI score > 6. Blood concentration was measured using high-performance liquid chromatography with fluorometric detection. Statistical analysis was performed using a nonlinear mixed-effect approach with the Monolix software. Results: A total of 168 blood samples were obtained from 55 pediatric patients. HCQ apparent blood clearance (CL/F) was dependent on patients’ bodyweight and platelet count. Patients with active cSLE had a lower mean blood HCQ concentration compared with inactive cSLE patients (536 ± 294 vs. 758 ± 490 ng/mL, *p* = 5 × 10^−6^). Among patients with HCQ blood concentration ≥750 ng/mL, 87.6% had inactive cSLE. Moreover, HCQ blood concentration was a significant predictor of disease status. Conclusion: We developed the first HCQ blood concentration–effect relationship for cSLE associated with active or non-active disease status. A prospective randomized study is necessary to confirm these results.

## 1. Introduction

Childhood-onset systemic lupus erythematosus (cSLE) is a chronic systemic autoimmune disease with incidence of 0.3–0.9 per 100,000 children-years. Compared with adults with SLE, children experience a greater disease activity and higher rates of renal disease [1,2,3]. In adults with SLE, hydroxychloroquine (HCQ) significantly decreases disease activity, with fewer relapses and improved damage-free survival [4,5]. Moreover, nonadherence to HCQ has been identified as a major cause of lupus flares [6,7,8]. Recently, it was recommended that all children with lupus should routinely be treated with HCQ [9]. 

After oral administration, HCQ is well absorbed with an absolute bioavailability of 79%. Peak blood concentrations are reached within 3 to 4 hours. HCQ is characterized by a large apparent volume of distribution (>2000 L) due to poor plasma protein binding (~50%) and high tissue binding, including blood cells [10]. The HCQ metabolic pathways involve cytochrome-P450s CYP2C8 and CYP3A and, to a lesser extent, CYP2D6 [10,11,12].

However, blood concentrations of HCQ show a high interindividual variability for the same administered dose. This variability has been observed in compliant adult patients with rheumatoid arthritis (RA), SLE, and COVID-19 [13,14,15,16,17]. A meta-analysis demonstrated the association between low HCQ levels and reported nonadherence and a threshold of 750 ng/mL blood HCQ concentration has been proposed as a target in the treatment of adults with SLE [16]. According to Chasset et al. when blood HCQ concentrations were higher than 750 ng/mL, a significant improvement of skin lesions was observed in patients with refractory cutaneous lupus erythematosus [18]. The relationship between blood concentrations of HCQ and clinical efficacy has also been demonstrated in RA [13,14]. However, no data are available in pediatric SLE. In this retrospective study, we aimed to investigate the relationship between HCQ blood concentration and SLE activity in order to optimize HCQ treatment of children with SLE.

## 2. Results

A total of 168 blood samples and 623 SLEDAI scores were obtained from 55 children, of which 94% were girls. Among the girls, 72% experienced menarche. There were 15 and 6 concentrations below the limit of quantification (BLQ) and detected as outliers, respectively, that were removed from the final analysis for nonadherence. Most subjects took 400 mg HCQ once a day (Table 1). The mean duration of HCQ treatment for patients without retinal toxicity was 3.1 ± 2.3 years. There were 56 and 1 observations corresponding to stage 2 and stage 3 chronic kidney disease (Schwartz < 90 and 60 mL/min/1.73 m^2^), respectively. There were 135 SLEDAI scores above 6 units (21.7%). The detailed patient characteristics are summarized in Table 1.

### 2.1. Data Analysis

Large interindividual variations in blood HCQ concentrations were observed. Blood clearance was significantly related to bodyweight (WT exponent was then fixed to 0.75 according to allometric scaling) and platelet count (PLAT). FFM was not a better descriptor for CL/F than total bodyweight. There was no significant effect of the before or after menarche status on CL/F. For these covariates, median physiologic values for an adult patient were used in the equation, 70 kg and 250 × 10^9^/L. The final covariate submodel for CL/F was then

CL/F (L/h) = 19.6 × (WT/70)^0.75^ × (PLAT/250,000)^−0.648^. (1)

**Figure 1:**
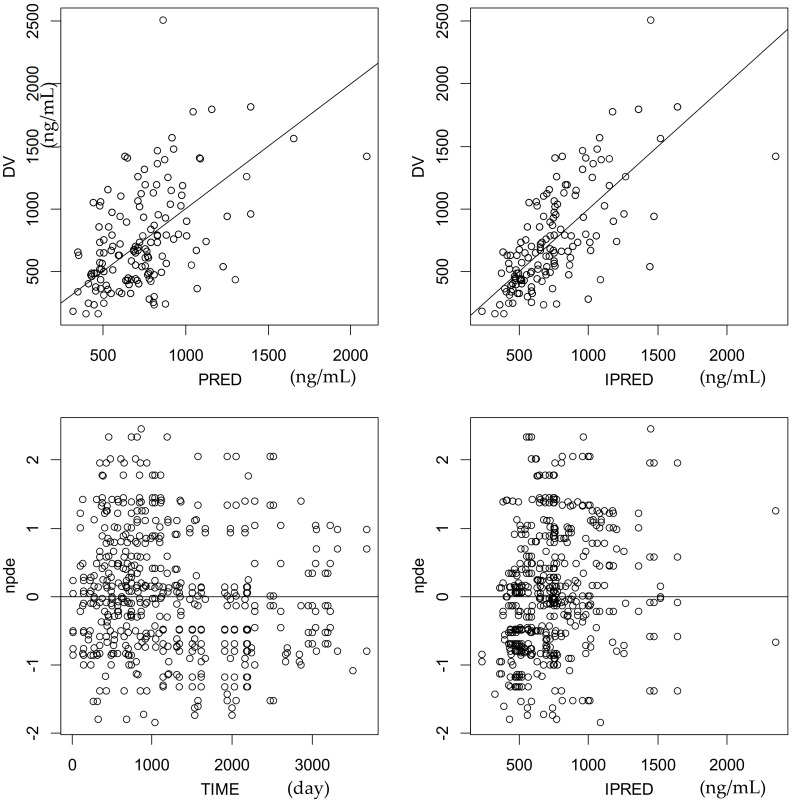
Goodness-of-fit plots showing the observed HCQ concentrations (DV) versus predicted (PRED) and individual predicted concentrations (IPRED) and normalized prediction distribution errors (npde) versus time and IPRED for the final model.

Parameter estimates are summarized in Table 2. The goodness-of-fit plots for the final model are shown in Figure 1.

### 2.2. Association between HCQ Treatment and SLEDAI Score

**Figure 2:**
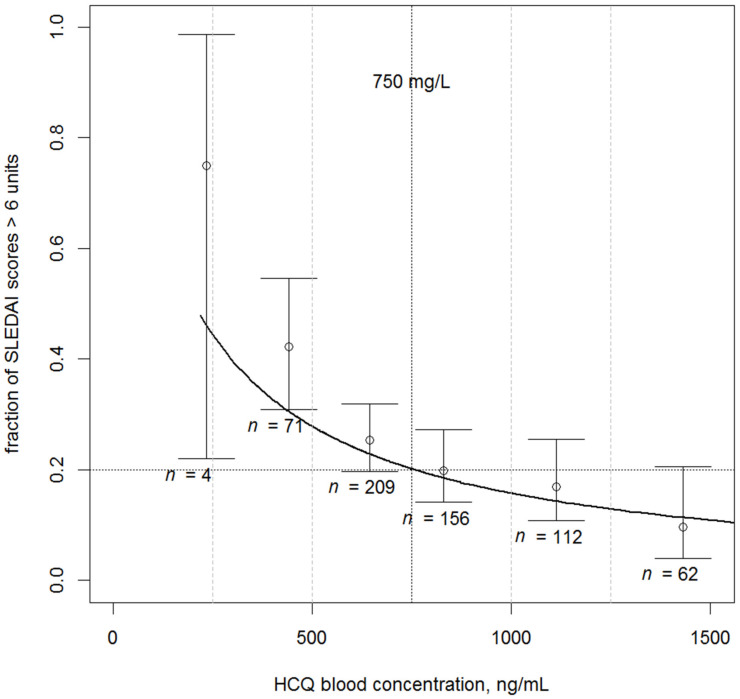
Observed and model-predicted percentages of scores > 6 units as a function of blood HCQ concentrations. Note that the 750 ng/mL threshold is associated with a 20% risk of active disease status. Open circles and segments, observed percentages with their 95% confidence intervals; black curve, median prediction; vertical dashed lines denote the concentration intervals for percentage estimations.

**Figure 3:**
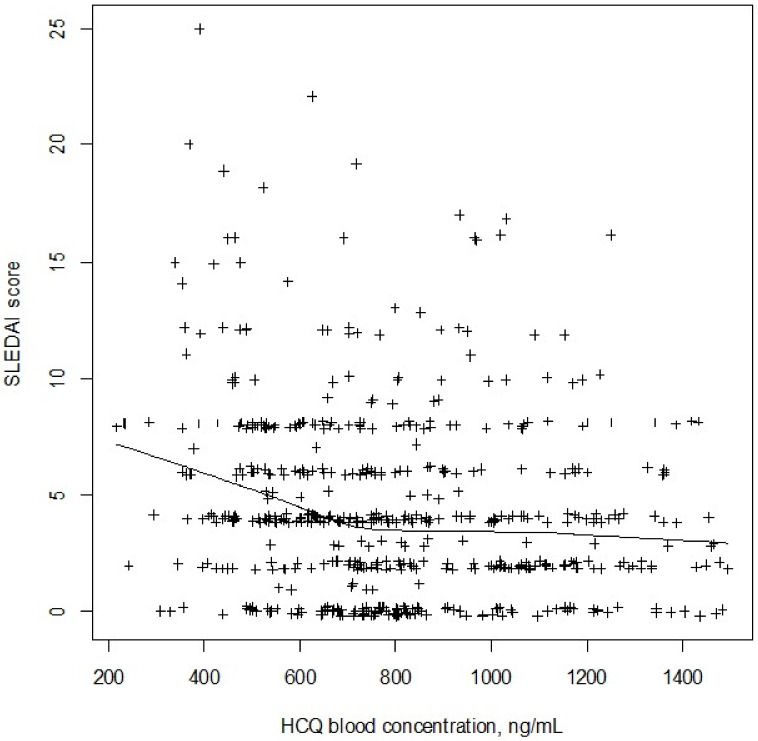
SLEDAI scores as a function of blood HCQ concentrations. The black curve is drawn after a spline function and shows the trend.

The mean SLEDAI score was 4.2 ± 3.9. Children with active cSLE had a lower mean C_HCQ_ than patients with inactive cSLE (536 ± 294 vs. 758 ± 490 ng/mL, *p* = 5 × 10^−6^). When C_HCQ_ was ≥750 ng/mL, 87.6% of patients had inactive cSLE. When C_HCQ_ was <750 or >750 ng/mL, an immunosuppressive treatment was present in 66.4% and 52.8% of patients, respectively (*p* = 0.0013). Response to HCQ treatment was significantly related to C_HCQ_. The treatment duration was not retained since the addition of this effect increased the BIC. The final logit relationship was

logit(P{SLEDAI > 6} = 5.55 − 2.41 × log_10_(C_HCQ_) (2)

showing that C_HCQ_ decreased the probability of active disease status. Figure 2 shows the decrease of the percentage of active disease cases versus C_HCQ_. Figure 3 shows the SLEDAI scores versus C_HCQ_. Note that the model prediction curve is included in the confidence intervals (CIs) of observed percentages. The odds ratio for the HCQ effect is then 0.09 (95% confidence interval 0.05–0.16), which indicates that one unit increase in one log10(C_HCQ_) unit results in a 9% decrease in the probability of active disease status.

### 2.3. Dosage Recommendations

**Figure 4:**
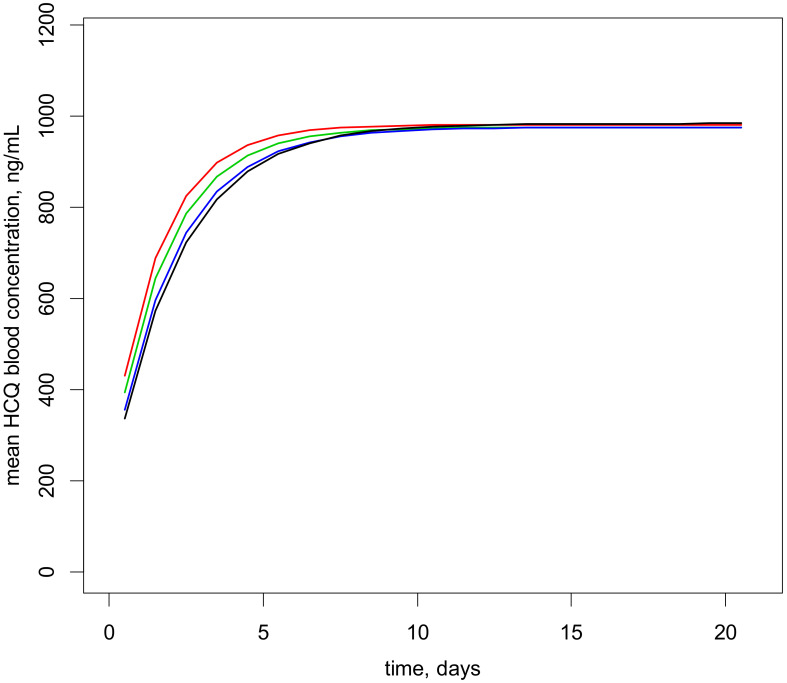
Mean blood HCQ concentration–time courses obtained using the dosing schedule from Table 3. Patient bodyweights are set to the mean of each weight band, 18 (red), 28 (green), 47.5 (blue), and 66 (black) kg. The curves connect the mean concentrations computed at each dose interval.

The dosing schedule to target blood HCQ concentrations > 750 ng/mL (therapeutic threshold) was derived from simulations of the final model. As summarized in Table 3, four weight bands from 15 to 70 kg were retained. This dosing schedule showed that approximately 45% were at risk of underdosage. Figure 4 depicts the mean concentration–time courses for these 4 weight bands, which reach the same plateau.

### 2.4. Blood HCQ Concentrations and Retinal Toxicity

Retinal toxicity was observed in three patients. These patients started treatment with HCQ at the age of 14.8, 15.9, and 8.8 years. The first patient received 200 mg of HCQ for 2.6 years then 400 mg for 1.2 years. For the other two patients, the dose was 400 and 200 mg for 0.8 and 8.9 years, respectively. Retinal toxicity was observed in 3 patients after 2.3, 0.8 and 8.2 years of treatment with HCQ, respectively. Their blood HCQ concentrations were <100, 199, and 459 ng/mL.

## 3. Discussion

This retrospective study shows that monitoring blood C_HCQ_ in cSLE might be useful to understand treatment failures and optimize treatment efficacy. Targeting HCQ levels ≥ 750 ng/mL appears to be a reasonable therapeutic threshold (80% of SLEDAI scores < 6) similarly to the recommended target in adult patients with SLE. Several studies have shown a correlation between C_HCQ_ and efficacy in SLE and rheumatoid arthritis in adults [14,15,16]. In this study, we demonstrated that bodyweight and platelet count significantly influenced HCQ blood clearance. The effect of weight was expected, since in this pediatric group, the weight ranged from 19 to 120 kg, and it is well established that HCQ CL is a function of WT0.75 [19]. Obesity did not significantly influence CL/F. However, with only 3 of 55 patients having a BMI > 30 kg/m^2^, the study lacked power to assess the influence of bodyweight on HCQ pharmacokinetics. The expression and activity of drug metabolism is affected by variety of physiological factors, (e.g., sex, menstrual cycle, age). Moreover, it has been demonstrated that estrogen downregulates CYP3A4 expression [20]. In our study, menarche had no significant influence on CL/F of HCQ. Platelet count also showed wide variations, ranging from 76 to 674 × 10^9^/L and the decrease of CL as a function of platelet count is likely due to HCQ binding to platelets, retaining HCQ in the bloodstream and thus limiting the distribution of HCQ to eliminating organs. High body mass index and low platelet count were significantly associated with low blood HCQ concentrations [11]. The CL/F estimation, 19.8 L/h/70 kg, which is very close to the value of 19 L/h per 69 kg or reported by Carmichael et al. [21] obtained using a similar method and 18.6 L/h per 53 kg reported by Morita et al. [12].

Moreover, blood HCQ concentrations were associated with disease activity, i.e., the higher the concentration of HCQ, the lower the percentage of active disease status. A similar relationship between low blood HCQ concentration and higher SLE activity was demonstrated in adult SLE [11]. Blood HCQ concentration was significantly higher in patients with inactive cSLE (758 ng/mL; range, 100–2509) than in patients with active cSLE (536 ng/mL; range, 50–1408) (*p* = 0.005).

Dosing recommendations for pediatric patients according to bodyweight could be determined. Considering a therapeutic threshold ≥ 750 ng/mL, a conservative dosing strategy was established in mg/kg/day for 4 bodyweight bands. Because of the high between-subject and residual variabilities observed with this orally administered drug, only 45–50% of the expected concentrations were in the window. Therapeutic drug monitoring should be applied to optimize HCQ dosage following 10–15 days of treatment. Furthermore, some of these dosage recommendations are higher than 6.5 mg/kg/day to reach the threshold of 750 ng/mL during the first month of treatment, and the tablet (200 mg) is not suitable for children weighing less than 25 kg. Therefore, a prospective analysis is necessary to confirm these results.

Limitations of this retrospective study: (i) compliance with therapy was mainly achieved using therapeutic drug monitoring; (ii) due to a relatively small sample size, no significant relationship could be found between blood HCQ concentration and retinal toxicity (in the three children with retinopathy, blood HCQ concentrations were <100, 199, and 459 ng/mL after 2.3, 0.8, and 8.2 years of treatment with HCQ, respectively). 

## 4. Materials and Methods

### 4.1. Patients and Drug Assay

In the present study, we performed a retrospective analysis of the requests of HCQ dosage collected in patients followed up in two French Reference National centers for pediatric rare autoimmune diseases between October 2009 and November 2018. Patients were enrolled if they fulfilled all the following criteria: disease that met the American College of Rheumatology classification criteria for SLE and diagnosed before 16 years of age; treatment with oral hydroxychloroquine sulfate (Plaquenil, Sanofi-Winthrop, Paris, France). The Schwartz equation was used for glomerular filtration rate (GFR) estimation. SLE status was assessed using the SLE Disease Activity Index (SLEDAI) at time of each HCQ dosage. Blood samplings were obtained during clinics, but the exact time of drug administration relative to blood sampling was not recorded. HCQ assay was routinely performed in the same pharmacological laboratory in order to assess patient compliance. All patients underwent an annual ophthalmological examination, comprising at least fundus examination, 10.2 automated visual field, and SD-OCT optical coherence tomography. Patients’ medical records were retrospectively reviewed for demographic, clinical, and biological characteristics and ophthalmological examination. Whole-blood HCQ concentrations were assayed by U-HPLC with fluorometric detection as previously described [22]. The study was performed in accordance with French regulations and approval was obtained from Necker Hospital Ethics Council (Ref 2018-NZ 7).

### 4.2. Data Analysis

According to pharmacokinetic principles, mean C_HCQ_ is related to apparent HCQ blood clearance (CL/F, where F is the unknown bioavailability) via the equation

C_HCQ_ × (CL/F) = Rate (3)

where Rate denotes the dose rate (Rate = dose/Interdose Interval). CL/F was adjusted to the bodyweight (WT) according to the allometric rule

CL/F = CL/F_POP_*(WT/70)^0.75^ (4)

The subscript POP denotes the average population value standardized for an adult weighing 70 kg.

Parameter estimation was performed using the non-linear mixed-effect modeling software Monolix (version 2019R2) that is able to estimate both between-subject (BSV or ω) and residual variabilities (σ). BSV was ascribed to an exponential model and σ to a proportional model. The observations below the limit of quantification (BLQ) were removed from the analysis because they were thought to indicate nonadherence. Observations more than 3 times or less than 1/3 of individual predicted concentrations were considered as outliers and removed from the final analysis. Individual characteristics that may influence CL/F were checked for bodyweight (WT), free-fat mass (FFM) REF [23], age, sex, renal function using the Schwartz index, platelet count, and serum albumin. For young females, the effect of menarche (before, after) was also investigated. The effect of quantitative covariates was modeled as shown in Equation (1). The Bayesian information criterion (BIC) was used to test for covariate effects. The goodness-of-fit of each model was evaluated by visual inspection of the observed versus population and individual predicted concentration, plus residual (npde, normalized prediction distribution error) scatter plots.

Given the zero inflation in the SLEDAI data, a linear regression of SLEDAI versus C_HCQ_ was not appropriate since the distribution deviated from normality. The SLEDAI scores were then converted to binary scores, inactive or active disease status, i.e., SLEDAI in 0 to 6 units or SLEDAI > 6 units [15]. The logistic regression expression was

Logit{P(SLEDAI > 6)} = b_0_ + b_i_ × log(V_i_) (5)

where the b_i_ terms denote the slopes quantifying the effects of explicative variables V_i_ denoted by subscripts i. For C_HCQ_, the values were set to the individual predictions that allowed to include all SLEDAI scores (using Monolix). 

Given the final results, 500 simulations of HCQ concentrations at steady state were performed for patient bodyweights ranging from 15 to 70 kg. The probabilities to observe concentrations greater than 750 ng/mL (risk of inefficacy) were then estimated.

## 5. Conclusions

Our study shows that blood concentrations of hydroxychloroquine are associated with disease activity in children with systemic lupus erythematosus. It suggests that blood hydroxychloroquine concentrations ≥ 750 ng/mL may be a potential therapeutic target concentration in this population.

## Figures and Tables

**Table 1:** Demographic, biological, and disease characteristics of the 55 patients.

Covariate	Mean ± Standard Deviation	Min	Max
Age, years	15 ± 2	5.5	18
Weight, kg	51 ± 18	18.8	120
Sex, (F) (*n*/*N*)	151/168	NA	NA
Schwartz, mL/min/1.73 m^2^	116 ± 26	34	201
Creatinine, µmol/L	50 ± 14	25	163
Albumin, g/L	38 ± 6	11	68
Hb, g/dL	12 ± 1.5	7	17.7
Platelet, 10^9^/L	267 ± 75	76	674
Proteinuria, g/L	1.2 ± 1.5	0	7.5
White blood cells, 10^9^/L	5.28 ± 2.1	2	10.8
C3 (mg/L)	887 ± 253	126	1460
SLEDAI score	4.4 ± 3.9	0	25
Dose HCQ, mg/kg/day	5.7 ± 2.1	2	15.7
Comedications:			
Corticosteroids (*n*/*N*)	136/168	NA	NA
Immunosuppressant (*n*/*N*)	104/168	NA	NA
Concentration HCQ, ng/mL	665 ± 433	100	2509
Sampling time, day	1100 ± 830	10	3674

NA: not applicable; Hb: hemoglobin; SLEDAI: Systemic Lupus Erythematosus Disease Activity Index; HCQ: hydroxychloroquine; n: number of cases; N: number of observations; C3: complement protein. All data were collected at the time of HCQ sampling.

**Table 2:** Population parameter estimates of HCQ kinetics and effects on disease status, active or non-active disease (SLEDAI score > 6 or not) in 55 children with systemic lupus disease.

Fixed Effects Parameters	Estimate	RSE (%)
Blood Clearance ^#^, CL/F_POP_ (L/h standardized to 70 kg)	19.6	5.35
Weight effect on CL/F, (WT/70)^WT^	0.75→fixed	NA
Platelet effect on CL/F, (Platelet/250,000)^PLAT^	−0.648	25
Effect on disease, SLEDAI score > 6 units ^##^		
intercept	5.55	30
b_HCQ_, slope for log_10_ (HCQ concentration) effect	−2.41	24
Variabilities		
ω_CL_	0.263	22
σ, proportional	0.385	8
ω_intercept_	1.88	18

^#^ For the ith subject, CL/F(WTi, Plat.counti) = 19.6 × (WTi/70)^0.75^ × (Platelet counti/250,000)^−0.648^; ^##^ logit(P(active disease)) = intercept + bHCQ*log10(C_HCQ_,ng/mL); rse (%): relative standard error; ω: between subject variability (log additive distribution; log(P) = log(PPOP) + ω); σ: proportional variability.

**Table 3:** Hydroxychloroquine dosage recommendation in mg/kg/day to achieve blood concentrations greater than 750 ng/mL (Prob: probability; C: blood HCQ concentration (ng/mL).

Bodyweight Band kg	Dosage mg/kg/Day	Prob (C > 750) ng/mL
15–21.9	9	53.4
22–34.9	8	55
35–60.9	7	54.4
61–71	6.5	52.7

## Data Availability

The data support the findings of this study are available on request from the corresponding author upon reasonable request.
